# The Feasibility and Acceptability of a Collaborative Deprescribing Intervention to Reduce Anticholinergic Burden Among Hospitalised Older Patients

**DOI:** 10.1111/bcpt.70277

**Published:** 2026-08-23

**Authors:** Kinda Ibrahim, Eloise Radcliffe, Edward Hewertson, Andrew Bates, Andy Fox, Emily Smith, Cathrine Mckenzie, Liam Jones, Sara Mckelvie, Ngianga Ii Kandala, Sara McCloskey, Tracey Sach, Stephen Lim

**Affiliations:** ^1^ Applied Research Collaboration Wessex National Institute of Health and Care Research (NIHR), University of Southampton Southampton UK; ^2^ School of Primary Care, Population Sciences and Medical Education, Primary Care Research Centre University of Southampton Southampton UK; ^3^ Medicine for Older People Department University Hospital Southampton NHS Foundation Trust Southampton UK; ^4^ Critical Care and Perioperative theme, NIHR Southampton Biomedical Research Centre, Faculty of Medicine University of Southampton Southampton UK; ^5^ Pharmacy Department NHS University Hospital Southampton Southampton UK; ^6^ Academic Geriatric Medicine NHS University Hospital Southampton Southampton UK

## Abstract

**Introduction:**

37%–48% of older patients receive anticholinergic medicines, increasing the risk for falls, delirium, dementia and death. Hospital admission may provide an opportunity for deprescribing to improve outcomes. This study evaluated the feasibility and acceptability of a digital tool to identify anticholinergic medicines and support deprescribing.

**Methods:**

A pre‐post feasibility study was conducted (May–September 2025). Patients aged ≥ 75 with an ACB score of ≥ 3, screened using a digital tool, were recruited. The identified medications were highlighted to the clinical team to prompt a medication review (stop, reduce or switch). Primary outcomes were recruitment and retention. Secondary outcomes included medication and patients’ outcomes, healthcare use and acceptability from qualitative interviews.

**Results:**

Recruitment was challenging; 65 out of 301 patients with ACB ≥ 3 (22%) were eligible, 21 participated, of which 15 (80%) completed follow‐up. Mean age was 84.7 years, and intervention costs were £9.33 per patient. Interviews indicated technical and organisational challenges. Mean ACB scores were 3.85 (admission), 3.45 (discharge), 3.0 (1 month) and 3.44 (3 months), with slight changes in cognition and delirium but no changes in depressive symptoms or quality of life.

**Conclusions:**

Although acceptable, recruitment and technical challenges limited feasibility. Findings should be interpreted cautiously due to the small sample size, and further refinement is needed before implementation.

## Lay Summary

Testing a Hospital Approach to Reduce Problematic Medicines in Older People.

This study tested a computer tool designed to help hospital doctors review medicines taken by older people. Some medicines can cause problems such as falls, confusion and memory difficulties, especially when several are taken together. Doctors used the tool to decide whether medicines could be stopped, reduced or changed. Twenty‐one patients aged 75 and over took part. This tool helped reduce the number of medicines that may cause these problems and led to some potential patient benefits. Patients and staff thought the approach was helpful and acceptable. However, there were difficulties recruiting people and problems with the computer system. More work is needed before the approach can be widely used in hospitals.

## 1 Background

Medications with anticholinergic properties are widely used in older adults to manage conditions such as depression, chronic pain, psychosis, urinary incontinence and allergic rhinitis, with an estimated 33%–47% receiving at least one such drug [1, 2]. This prevalence is expected to rise with increasing multimorbidity and polypharmacy [3]. Anticholinergic activity is associated with a range of adverse effects, including confusion, cognitive impairment and parasympathetic symptoms such as dry mouth and constipation [4]. Anticholinergic burden—defined as the cumulative effect of taking one or more medicines with anticholinergic activity—has been linked to numerous adverse outcomes in older people, including impaired physical function, higher falls risk, cognitive decline, dementia and increased all‐cause mortality [5, 6, 7, 8].

Multiple tools have been developed to quantify anticholinergic burden, each categorising medicines according to their anticholinergic potency [4]. However, there is no consensus on a preferred tool for patient‐level assessment, and existing scales vary considerably in both drug inclusion and scoring approaches [9]. A recent review identified 21 anticholinergic burden scales, with substantial heterogeneity in drug coverage (ranging from 27 to 217 medications) and inconsistent scoring of 74% of high‐potency drugs [4]. Variability in development methods, geographical context and consideration of factors such as dosage further limits their consistency and clinical applicability, contributing to inconsistent associations with clinical outcomes. As a result, no gold standard tool has been established, highlighting the need for a standardised, clinically relevant approach that accounts for cumulative exposure and individual patient factors.

Despite guideline recommendations to review medications and assess anticholinergic burden in older adults presenting with falls or delirium [10, 11], approximately half of older adults continue to receive at least one anticholinergic medication [3]. This burden disproportionately affects vulnerable groups, including women and those from lower socioeconomic backgrounds [3]. In practice, the clinical utility of anticholinergic burden tools is further limited by low awareness and confidence among clinicians, with only 37% of UK clinicians reporting the ability to accurately assess anticholinergic burden [12]. Evidence on prescribing patterns and deprescribing practices in hospital settings remains limited. An international observational study found no reduction in anticholinergic burden during acute hospital admissions among older adults with delirium, cognitive impairment or falls, suggesting missed opportunities for optimisation [13].

Despite growing recognition of the harms associated with anticholinergic burden, there remains a lack of evidence on scalable approaches to systematically identify and reduce anticholinergic prescribing among hospitalised older adults. A recent review identified key barriers, including limited multidisciplinary collaboration, low prescriber confidence and resource constraints, alongside facilitators such as pharmacist involvement and the use of digital decision‐support tools [14]. However, most intervention studies have been conducted in community or primary care settings [15]. A recent systematic review focusing on hospitalised older adults identified only eight, predominantly low‐quality studies, largely centred on pharmacist‐led medication reviews, sometimes combined with education or stewardship approaches [16]. Although these interventions demonstrated improvements in prescribing outcomes and high acceptance of recommendations, evidence regarding safety, cost‐effectiveness and clinical outcomes remains limited. Existing hospital‐based interventions have largely focused on pharmacist‐led medication reviews, with limited evidence on digitally enabled approaches that can systematically identify patients with high anticholinergic burden and support deprescribing within routine clinical workflows. This study addresses that gap by evaluating the feasibility and acceptability of a digitally supported deprescribing intervention in acute care.

## 2 Methods

### 2.1 Study Design and Setting

This was a nonrandomised pre‐post–quasiexperimental study testing the feasibility of implementing a novel deprescribing intervention on Medicine for Older People (MOP) wards at the University Hospital Southampton (140 beds), which admit unselected emergency medical patients aged 75 years and over with multimorbidity. The average length of stay among this population is 7 days. This study was registered on ClinicalTrials.gov (Record DART351020) and received ethical approval from the UK Health Research Authority (Research Ethics Committee reference: 25/LO/0171).

The study was conducted in accordance with the Basic & Clinical Pharmacology & Toxicology policy for experimental and clinical studies [17]. The results of the study are reported in accordance with the Strengthening the Reporting of Observational Studies in Epidemiology (STROBE) Statement: guidelines for reporting observational studies [18] (see Appendix S1, supplementary file).

### 2.2 Intervention

A computer‐based web application is used in the hospital to visualise data to aid specific medicines management issues. A digital tool designed to identify anticholinergics was developed to provide patient‐level information on anticholinergic medications. Anticholinergic burden score was calculated for each patient by estimating the extent to which an individual patient may be at risk of anticholinergic adverse effects by ranking medications on a 3‐point scale from 0, *No or low risk* to 3, *High anticholinergic potential*. The digital tool generates, for each patient, the total anticholinergic burden score and displays the names of individual drugs contributing to that risk with their individual risk score. The scores used incorporated three validated lists: Anticholinergic Effects on Cognition (AEC), NHS polypharmacy scale and ACB calculator relevant to the UK market to capture the most available opportunities to benefit patients by deprescribing. When a medication had a different score on more than one scale, the highest score was used. A snapshot audit for baseline data of 96 patients has shown that 30% of patients who are admitted to the study wards had an anticholinergic burden score > 3 and 27% of them still had a score of > 3 on discharge. This demonstrated a significant opportunity for deprescribing that has not been achieved with standard care.

The intervention was developed and agreed in the preparation of this proposal through multiple stakeholder meetings with the geriatric medicine consultants and the pharmacy team in MOP. The intervention included the following elements:

1. A pharmacist or resident doctor uses the digital tool (2–3 times weekly) on the study wards to identify patients admitted with anticholinergic burden score of 3 or more.
2. A pharmacist or resident doctor highlights those patients to the geriatrics team by placing a sticker note in patients' medical records that details the total anticholinergic burden score for the patient and the individual medications contributing to that score with their individual scores to encourage consultants/doctors to consider deprescribing these medications.
3. The sticker acted as a prompt for clinicians to review the patient's medications and consider whether anticholinergic medicines should be stopped, reduced or switched based on clinical judgement and patient circumstances.
4. Any medication change is highlighted in the discharge letters to general practitioners (GPs) including any plans for tapering medications after discharge.

### 2.3 Participants and Recruitment

Eligible patients included those aged ≥ 75 years admitted to one of the five MOP wards, who have an ACB score of 3 or more, and are able to provide informed consent. Those who were expected to have a limited life expectancy, receiving end‐of‐life care, have dementia or are unable to consent were excluded. Eligible patients were then approached by the research delivery team who explained the study and invited them to take part. A written consent form was then completed with those who agreed to participate, and baseline data collection was completed.

### 2.4 Sample Size

Recruitment ran between May and November 2025, with the aim of recruiting 50 patients in total. No formal sample size calculation has been performed. However, with 50 patients it would be possible to estimate a proportion‐based outcome measure with sufficient precision to compute a confidence interval to within 0.15 either side of an estimated proportion of 0.50 (i.e., 25 out of 50) at a two‐sided 95% level of significance.

### 2.5 Data Collection and Outcomes

We collected outcome measures from patients using questionnaires at two time points: At baseline (T0) in‐person although an inpatient and at 3‐month follow‐up (T4) after discharge either in‐person or over the telephone. Data at baseline included patients' demographics (age, gender, level of educational attainment, marital status, usual residence and ethnicity), number of comorbidities and cognition using the Abbreviated Mental Test Score (AMTS) [19]. We also collected the following outcomes at baseline and 3‐month follow‐up using validated measures: Functional outcomes using the modified Barthel index for activities of daily living [20], frailty using the Clinical Frailty Score (CFS) [21], depression using the Geriatric Depression Scale (GDS) [22], delirium using the 4AT (a simple and short (< 2 min) delirium detection tool designed for easy and effective clinical use) and quality of life (QoL) with EQ‐5D‐5L [23].

The Care and Health Information Exchange (CHIE) database was used to obtain routinely collected data on medication and health care use. It is a secure, specialised NHS database designed for analysing population health trends to improve patient care, service planning and research. Routinely collected data such as medications were extracted from patient medical records upon patients' consent at admission (T1), discharge (T2), 1 month postdischarge (T3) and 3‐month follow‐up postdischarge (T4). The number and therapeutic classes of medications included in the British National Formulary (BNF) Chapters 1–4 and 6–10 [24] were recorded which are used for chronic conditions excluding topical and acute medications. Focusing on these medications has been recommended in several systematic reviews [25, 26]. Anticholinergic burden scores were calculated for each patient at the four time points using the digital tool.

**The primary outcomes** include feasibility outcomes such as the following.

- The proportion of eligible patients who agreed to take part in the study (recruitment rates);
- Follow‐up rates;
- Intervention fidelity was assessed by recording the proportion of patients identified by the digital tool who subsequently had a sticker placed in their medical records;
- Medication‐related outcomes including total number of medications at each time point, anticholinergic scores at each time point and types of anticholinergic medications;
- Completion of health and patient‐related outcome measures (e.g., SARC‐F, modified Barthel index, GAD).

**Secondary outcomes**

**Health economics:** Healthcare resource use data (primary, secondary and community care resource use, as well as medication use) was collected through questionnaires at baseline and 3‐month follow‐up. Health‐related quality of life was measured using the EUROQOL EQ‐5D‐5L instrument [27].

Acceptability outcomes: Interviews were conducted at the end of the feasibility study with a purposive sample of healthcare professionals who were involved in delivering the intervention, and patients and carers who received the intervention. Topic guides were codeveloped with the study patient and public involvement group to explore barriers and facilitators to implementation, explore the views of those involved and refine and optimise the intervention design prior to a larger trial (see Appendix S3, supplementary file). All interviews were recorded and transcribed verbatim, with any identifying data anonymised.

### 2.6 Analysis

The number and characteristics of participants recruited were summarised using descriptive statistics. To keep participant information private, we followed statistical disclosure control principles. We combined categories with small cell counts (fewer than 5) and made sure to either suppress or summarise any potentially identifiable details. Percentages are rounded and presented in aggregate form in accordance with statistical disclosure control requirements. For categorical data, results are reported as frequencies and proportions, whereas for continuous data, the mean (SD), mean difference and their associated 95% confidence intervals are presented. Outcome measures including CFS (frailty), SARC‐F (sarcopenia) and EQ 5D (quality of life) are reported first on their continuous scale, providing full distributional information, and then categorised using definitions and cut‐off points to aid interpretability. Prior to the analysis, the distribution of changes in continuous outcome measures at baseline and after 3 months was assessed, and paired *t*‐tests were utilised. All statistical tests were two‐sided, employing a 5% significance level (*p* < 0.05). As the primary aim of this feasibility study is not to assess statistical significance, 95% confidence intervals/*p*‐values are presented merely for reference, to support the interpretation of trends. All analyses were conducted using SAS software, version 9.4 (SAS Institute Inc., Cary, NC, USA).

Qualitative data were analysed using inductive thematic analysis in an iterative way, with the assistance of Large Language Models (LLMs) for qualitative analysis, as a supportive analytical tool to assist with initial coding and theme generation rather than as an autonomous analyst [28]. After the researchers (ER and KI) familiarised themselves with the data, the tool (M365 Copilot, based on the GPT‐5 chat model) was used to code the data using Braun and Clarke's thematic analysis and pull‐out emergent themes with supporting quotes [29]. An initial prompt was used asking for each transcript to be coded using Braun and Clarke's thematic analysis to explore acceptability of the DART intervention, providing supporting quotes. Further prompts were used for more detailed analysis and to request further example quotes from the transcripts. Each transcript was analysed separately and then a prompt was used to compare themes across transcripts, with follow‐up prompts used for further in‐depth comparison and development of themes, using an iterative approach. All generated codes and themes were reviewed, checked against the original transcripts and refined by two researchers through iterative discussion, with final interpretation remaining the responsibility of the research team. Ethical guidelines associated with use of AI in research are limited and evolving, so no standardised guidance currently exists in the United Kingdom. All usual ethical regulations were followed, and special measures were taken to ensure data privacy and security, including the use of a secure, GDPR‐compliant AI tool, which does not use research data as training material [28].

Health economics: Completeness was explored for all measures of resource use, medication use and quality of life. Resource use was valued according to published NHS and PSSRU 2023/2024 unit costs [30]. Medications were valued according to the mean price of all medications listed on the Prescription Cost Analysis (PCA) [31]. The cost of the intervention was estimated using PSSRU unit costs applied to the average time it would take a pharmacist or registrar medical doctor to complete the intervention (see Appendix S1 for unit costs). Utility values were estimated from the EQ‐5D‐5L using the approach recommended by NICE at the time of analysis and quality adjusted life years (QALYs) were estimated over the 3‐month period using linear interpolation and area under the curve without adjustment [32]. Mean (SD) for all measures is reported along with the mean (95% CI) difference in resource use and costs comparing baseline to 3 months. The health economic results are presented as a descriptive analysis because their purpose was to help inform the design of a future economic evaluation (see Appendix S2, supplementary file for detailed analysis).

## 3 Results

### 3.1 Feasibility Outcomes

**FIGURE 1:**
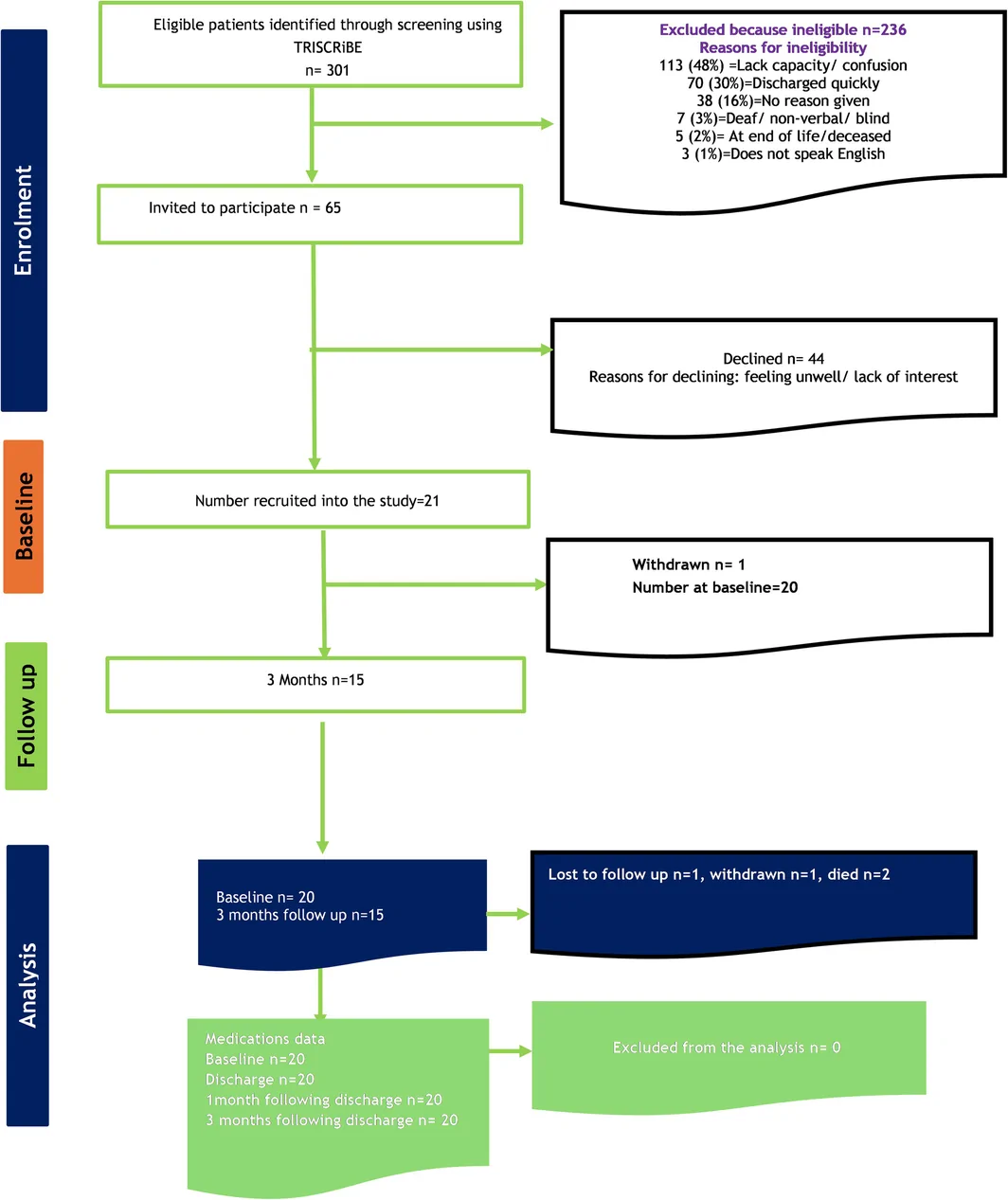
Consort diagram of the study.

A total of 301 patients were identified during the study period to have an ACB score ≥ 3 ranging between 3 and 7 (see Figure 1). Two hundred seventy‐five patients (91%) had a sticker in their note to notify the team of patient ACB score and highlight the specific medications. A total of 236 (78%) were identified ineligible to consent (lack capacity to consent), with almost 50% lacking capacity and 28% discharged quickly highlighting recruitment challenges. Of the 301 identified patients, only 65 were deemed eligible (22%) to consent and were approached by the research team. However, 44/65 declined participation, and only 21 (out of the target sample of 50 patients) took part in the study (recruitment rate); however, one withdrew before completing baseline data, suggesting feasibility issues. During the 3‐month follow‐up, two patients died, another withdrew, and one was lost to follow up. Follow‐up rate was 15/21 (80%).

**FIGURE 2:**
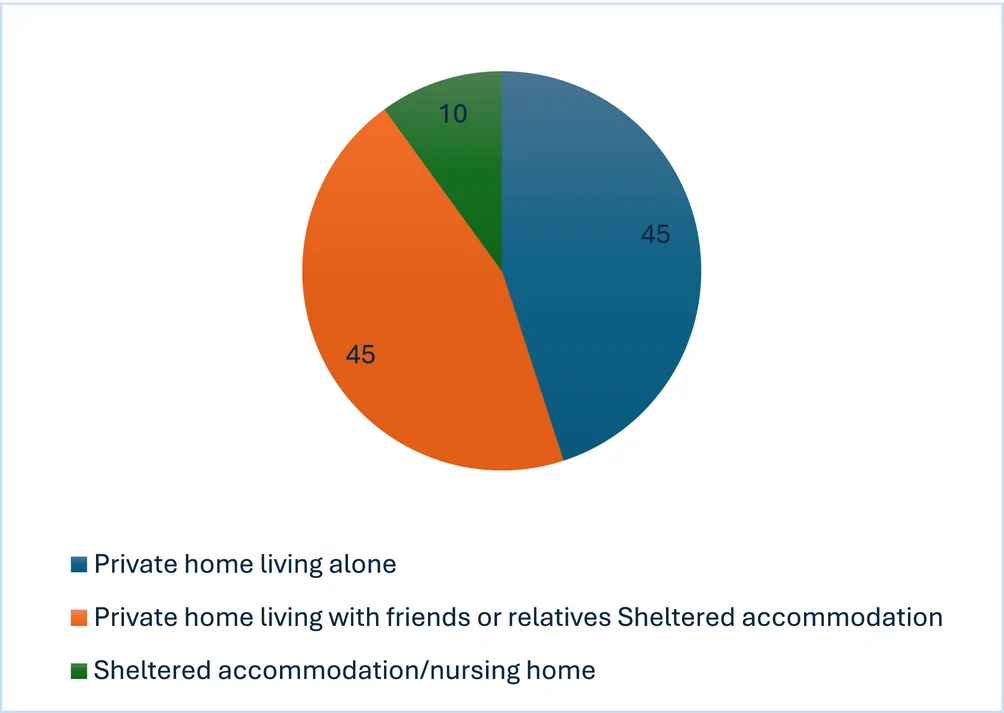
Percentage distribution of participants living arrangement.

**FIGURE 3:**
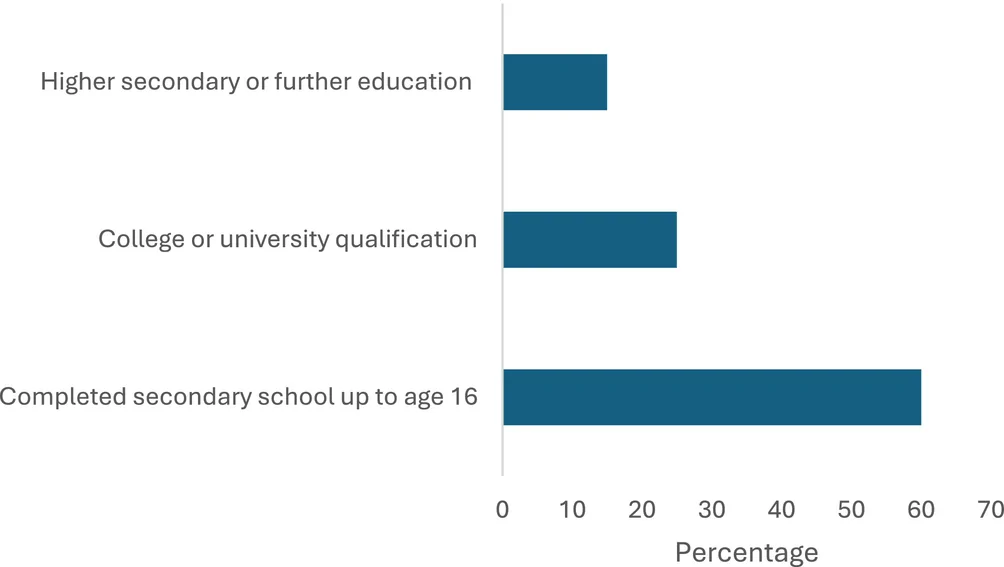
Percentage distribution of participants education level.

An overview of participants' characteristics at baseline is presented in Table 1. The participants mean (SD) age was 84.7(4.24) years (Range 75–92) with 55% female participants. Most of the participants were married, nearly two‐thirds (65%), with 35% single and 45% lived at home either alone or with family or friends (Figure 2). The majority (60%) reported completing secondary school up to age 16. A further 25% had attained college or university qualification, whereas 15% had completed higher secondary or further education (Figure 3). The mean number of long‐term medications was 9.4 (±6.25) and the mean number of medications was 12.05(±4.15) ranging between 6 and 25. At baseline 55% of patients were frail (CFS), 70% were at risk of sarcopenia (SARC‐F), 45% of patients were moderately or severely dependant (Barthel), 75% patients were classified as living with possible deliruim (4AT), and 65% had depression or potential depression (GDS).

**TABLE 1:** Participants baseline characteristics.

Variable	Number (*n*)	Percentage (%)
Gender		
Female	11	55.0
Male	9	45.0
Age in years, mean (SD) [min–max]	84.7(4.24) [75–92]	
Marital status		
Single	7	35.0
Married	13	65.0
Number of comorbidities mean (SD) [min–max]	9.4(6.3)	[2–20]
Number of medications mean (SD) [min–max]	12.05(4.2)	[6–25]
Abbreviated mental test (AMTS) mean (SD) [min–max]	8.‐5(1.3‐)	[6–10]

Abbreviations: max, maximum value; min, minimum value; SD, standard deviation.

Collecting health and patient‐related outcome measures (e.g., SARC‐F and modified Barthel index) was feasible and complete from all patients at both baseline and 3‐month follow‐up. As shown in Table 2, there was no statistically significant change in depression, function and quality of life scores between baseline and follow‐up. However, both delirium and AMTSs changed slightly at 3 months. At baseline 85% patients had at least one hospital admission in the previous 3 months. 50% patients had at least one hospital admissions in the 3 months following discharge.

**TABLE 2:** Means outcome measures at baseline and 3‐month follow‐up.

Variable	Baseline (*n* = 20)	At 3 months (*n* = 15)	Means difference (BL‐3 M)	*p*
			Mean (95% CI)	
SARC‐F	5.4	4.7	1.4 (0.2, 2.6)	**0.025**
Barthel	87.8	72.3	14.4 (0.57, 28.2)	0.042
Geriatric Depression Scale (GDS)	5.5	5.3	0.4 (−1.02,1.8)	0.554
4AT (delirium)	1.2	0.7	0.53 (0.07, 1.0)	**0.027**
AMTS	8.5	9.3	−0.87 (−1.37, −0.4)	**0.003**

Abbreviations: 3 M‐3 months; 4AT; arousal, attention, abbreviated mental test—4 and Acute change; AMTS, Abbreviated mental test score; BL, baseline; CI, confidence interval; SRAC‐F, strength, assistance with walking, rising from a chair, climbing stairs and falls.

### 3.2 Medication‐Related Outcomes

The mean number of medications included in the BNF Chapters 1–4 and 6–10 was 12.05(±4.2) at admission, 11.95(±4.3) at discharge, 11(±4.5) at 1‐month follow‐up and 11.5 (±4.4) at 3‐month follow‐up.

Mean (SD) ACB scores were 3.9(±1.9) at admission, 3.5(±2.2) at discharge, 3(±2.1) at 1 month and 3.4(±2.2) at 3 months. The most commonly prescribed medications with anticholinergic effects included opioids (*n* = 10 ACB 1), furosemide (*n* = 9; ACB 1), amitriptyline (*n* = 6; ACB 3), prednisolone (*n* = 6, ACB 1), aspirin (*n* = 6, ACB 1), digoxin (*n* = 4, ACB 1) and solifenacin (*n* = 4, ACB 3), and two were taking loratadine (*n* = 2, ACB 1). Additionally, one patient each was prescribed warfarin, theophylline, tolterodine and hydralazine (*n* = 1 each, ACB 1).

### 3.3 Health Economic Data

The intervention was estimated to take 10 min to administer. This cost £9.33 if completed by a registrar grade medical doctor and £9.17 if completed by a pharmacist.

All participants had complete resource use data at baseline. One of the remaining 15 participants had missing data for all resource use items at 3 months. There were no significant differences in resource use or costs reported at each time point. The most frequently reported items included hospitalisations, GP visits, nurse visits and ‘other’ community care. Many resource use items were not reported in the study, indicating that it might be feasible to simplify the resource use form in a future trial. Medication data could not be costed individually because data on the duration of use was not gathered during the trial. In a full trial this extra detail should be included. Details on the unit costs, mean resource use, mean costs and mean medication use and mean medication costs can be found in supplementary file.

The EQ‐5D‐5L was completed by all participants at baseline and all the 15 remaining participants at 3 months. Twenty and 15 unique health states were reported at baseline and 3 months, respectively. Mean (SD) utility was 0.587 (0.217) at baseline and 0.489 (0.272) at 3 months. Mean (SD) unadjusted QALYs across the 3‐month period were 0.128 (0.054), equating to 51.2% of perfect health over the period. More details on the EQ‐5D data can be found in Appendices 6 and 7.

### 3.4 Acceptability of the Intervention

Qualitative interviews were conducted with four patients, one carer (a wife) and four healthcare professionals involved in the intervention implementation.

### 3.5 Recruitment and Research Delivery Challenges

Recruitment and data collection were affected by the complexities of conducting research in an acutely unwell, older inpatient population. Initial recruitment was limited by restrictive eligibility criteria, including the exclusion of patients lacking capacity, with later amendments perceived as beneficial but implemented too late to substantially improve recruitment. The original 24‐h consent window was also considered impractical in the context of short hospital stays and weekend staffing constraints. Adjustments to increase flexibility were viewed positively.

‘This age group … already have much going on … in the hospital, acutely ill … Maybe if this was done at the primary care level … they would be received in a different way’. (HCP 3)

Additional challenges included sensory impairments, illness severity and patient fatigue, which limited participation. Some outcome measures, such as the Geriatric Depression Scale, were reported to cause discomfort or distress, particularly in a ward environment lacking privacy. These findings highlight the need for flexible, patient‐centred research processes and careful selection of outcome measures in this population.

### 3.6 System and Workflow Barriers to Implementation

Significant system‐level and workflow challenges limited the consistent delivery of the intervention. Although the digital tool was perceived as having strong potential, its functionality was undermined by technical failures, including missing patient data and periods of prolonged downtime. In addition, poor integration with existing hospital systems and lack of alignment with clinical workflows reduced its usability in practice. This is highlighted in the following quote.

Patients' details … were just not pulling through …. There were quite a few breaks in the recruitment process … because of these technical difficulties. (HCP 4)

Screening and intervention processes were also described as time‐consuming and logistically challenging. Difficulties accessing patient notes, particularly in busy ward environments, and reliance on paper‐based systems (e.g., green stickers) contributed to missed opportunities and duplication of work. There was no systematic mechanism to confirm whether clinical prompts had been reviewed or acted upon, limiting intervention fidelity.

There would not be documentation afterwards … [verifying] the information was seen by the doctors … maybe we could have an extra step … to see if they've actually made a review or … decide to ignore it. (HCP 3)

Participants highlighted the need for improved digital integration, such as electronic flagging systems, and more streamlined processes to support routine implementation.

### 3.7 Workforce Capacity and Role Optimisation

Workforce constraints and role allocation were key determinants of implementation. Although pharmacy‐led screening was originally intended and widely regarded as the optimal model, this was not feasible due to workload pressures. Healthcare professionals consistently highlighted the expertise of pharmacists in medication optimisation and suggested that greater pharmacy involvement could improve both efficiency and quality of screening as illustrated in the following quotes:

Involvement of stakeholders such as pharmacists would be useful … .A lot of our pharmacists are incredibly experienced and actually suggest these changes or make these changes themselves. (HCP 2)

Initially we wanted to do [the screening] with pharmacy and I think it would make much sense … they have … better access to it and … more time … … if [the screening] was done more often, we would have higher numbers. (HCP3)

In practice, screening was undertaken by registrars, whose limited time and rotational roles created challenges for continuity and consistency. Participants noted that clearer delineation of responsibilities within multidisciplinary teams, alongside protected time for intervention‐related tasks, would enhance delivery.

These findings emphasise the importance of aligning intervention design with workforce capacity and leveraging the most appropriate professional skill sets.

### 3.8 Knowledge, Confidence and Decision‐Making in Deprescribing

Variation in prescriber knowledge and confidence influenced how the intervention was enacted. Responses to clinical prompts (e.g., green stickers) were inconsistent, with some clinicians making rapid or multiple medication changes, whereas others were more cautious or reluctant to act.

Resident medical doctors, in particular, were perceived as making ‘unilateral’ changes to medicines in response to the green sticker but were felt to require further training in anticholinergic burden and deprescribing principles. There was also hesitancy to modify medications initiated by specialist teams, particularly in areas such as mental health, reflecting perceived clinical risk and professional boundaries.

Some junior doctors may have interpreted the green sticker as an instruction to be acted on ‘unilaterally’ which possibly ‘prompted too many changes too quickly, and a sort of small gradual approach would have been better’ (HCP 1).

Participants identified a need for additional training and decision‐support tools, including concise guidance on anticholinergic burden, deprescribing strategies and medication tapering. Suggested innovations included embedding educational resources (e.g., QR codes) within the intervention to support real‐time decision‐making.

### 3.9 Communication, Patient Experience and Continuity of Care

Patients' ability to understand and recall medication decisions was significantly limited by the context of acute illness and the hospital environment. Many described how multiple admissions and interactions with different clinicians blurred together, making it difficult to distinguish specific conversations: ‘I very rarely saw the same doctor twice’ (Patient 17). This fragmentation was compounded by the physical and cognitive strain of being unwell, alongside the distractions of a busy ward, where ‘things are going on … you don't take everything in’ (Patient 17). As a result, patients often had only partial or absent recall of medication changes:

I had been taking omeprazole and they've taken me off that … don't ask me why … [the doctor] did mention something (Patient 4).

Within this context, patients tended to adopt a passive role in decision‐making. Although some expressed anxiety or uncertainty, this was typically overridden by a strong sense of trust in clinicians and a perceived lack of expertise:

I just have to accept what is recommended … I don't know one [medicine] from t'other really. (Patient 8)

You just have to trust them, don't you. (Patient 4)

This passivity was reinforced by reluctance to question staff—patients often did not want to ‘trouble’ busy healthcare professionals or felt too overwhelmed to engage.

Both patients and healthcare professionals identified written information as a potential strategy to mitigate these challenges. Patients and carers highlighted the value of accessible resources that could be revisited after conversations:

If you don't understand why you're given this medicine please ask the doctor … I think it would be helpful. (Carer 13)

Similarly, healthcare professionals emphasised the need for tools that clearly communicate risks and benefits as such resources may help reduce reliance on memory and support more active engagement:

Having a resource to refer patients to … something that communicates risks and benefits … would be helpful. (HCP 4).

The role of family carers emerged as another critical factor. Carers frequently supported medication management at home, particularly for patients with physical or cognitive limitations, yet were not consistently included in hospital‐based decision‐making. This gap was often attributed to logistical barriers, such as restricted visiting times, despite the importance of their involvement for continuity of care.

Finally, system‐level communication gaps further constrained patient understanding and continuity. Patients described instances where inadequate explanation of medication changes led to negative consequences, including symptom deterioration and the need for represcribing in primary care. Across interviews, there was a clear reliance on general practitioners and community pharmacists to provide clarification postdischarge, reflecting broader challenges in communication between secondary and primary care. Delays in discharge summaries and limited documentation of medication changes exacerbated this issue.

## 4 Discussion

This feasibility study examined the implementation of a deprescribing intervention supported by a digital tool to reduce anticholinergic burden among hospitalised older adults. Feasibility issues within the study were identified as a result of recruitment challenges due to technical issues and the high proportion of ineligible patients (mainly owing to cognitive impairment or rapid discharge). However, those who were able to participate engaged well, with good follow‐up completion and acceptability. A small decrease in the number of medications and anticholinergic burden score, as well as slight changes in delirium and cognition scores, were observed at 3‐month follow‐up. However, due to the small sample size, it is not possible to interpret these results or draw definitive conclusions, consistent with guidance on feasibility studies, which emphasises their exploratory nature [33]. In addition, the findings highlight how cognitive overload, environmental pressures and fragmented communication systems collectively limit patient engagement, reinforcing passive trust in clinicians while undermining understanding. Previous research has shown that hospitalised patients often have limited recall of medication discussions due to illness severity and environmental complexity [34]. Addressing these challenges requires not only improved interpersonal communication but also structural solutions such as clear written information, better inclusion of carers and strengthened integration between care settings to support patients in navigating medication decisions more effectively [35].

The study identified a substantial number of older inpatients with high anticholinergic burden (ACB ≥ 3), confirming that opportunities for medicines optimisation in this population are frequent and underaddressed. High anticholinergic burden has consistently been associated with adverse outcomes, including cognitive impairment, delirium and increased risk of falls [36]. In our study, several anticholinergic medications used in older people have weak individual effects (score = 1), such as prednisolone, furosemide and aspirin; however, their cumulative contribution to overall burden is clinically significant [37]. Chronic use of opioids such as codeine or morphine—although associated with low anticholinergic scores, but centrally reducing acetylcholine—contributes to sedation, delirium, constipation and falls, making them important targets for deprescribing, particularly when used long term without clear indication [38]. Other stronger anticholinergic medications identified in the study cohort included amitriptyline and solifenacin. Amitriptyline is recognised as a high‐risk medication due to its strong anticholinergic and sedative effects and is listed as potentially inappropriate in older adults [39]. These effects are associated with cognitive impairment, orthostatic hypotension, confusion and increased falls. Guidelines recommend reviewing its use and switching to safer alternatives where possible. Solifenacin, used for overactive bladder, also carries a high anticholinergic burden and has been associated with an increased risk of cognitive impairment and dementia in older adults [39]. Reducing exposure can involve dose reduction, deprescribing or switching to lower burden options such as mirabegron, alongside nonpharmacological strategies including bladder training and pelvic floor therapy.

The intervention prompted clinical teams to review high‐risk medications, reflected in modest reductions in anticholinergic burden. This suggests potential for clinically meaningful change if supported by ongoing stewardship, closer integration with primary care or reinforcement mechanisms beyond discharge. Evidence suggests that effective deprescribing interventions typically require structured, multidisciplinary approaches and ongoing follow‐up to sustain benefits [40]. The study also highlights important implementation considerations within busy acute care environments. Competing clinical priorities, workforce pressures and limited pharmacist capacity affected the consistency with which the intervention could be delivered, emphasising the need for adequate resourcing and clearer integration into routine workflows in future studies. Similar barriers have been reported in previous studies of hospital‐based deprescribing, particularly relating to time constraints and role clarity among healthcare professionals [41]. Although screening was originally intended to be undertaken by the pharmacy team to prompt medication review, workload constraints meant this responsibility shifted to resident medical doctors and registrars, which may have implications for intervention fidelity and sustainability. In addition, the reliance on manual, paper‐based prompts and limited integration with existing hospital systems further constrained consistency and scalability. Future iterations of the intervention should prioritise full integration with electronic health records, including automated alert systems to support identification and review of high‐risk medications. Such digital enhancements are likely to improve reliability, workflow integration and overall fidelity and will be critical to achieving scalable and sustainable implementation in routine care.

Strengthening multidisciplinary collaboration and continuity between hospital teams and primary care is essential to support sustained deprescribing after discharge and reduce the risk of medication reinitiation, as previous research demonstrates that successful deprescribing relies on coordinated approaches, clear communication and shared ownership of decisions across care settings [42, 43]. Embedding decision‐support processes within routine pathways, alongside sufficient workforce capacity, may further enhance the scalability and effectiveness of interventions. At the same time, improving communication with patients and carers is critical, as high cognitive load, the busy ward environment, interactions with multiple healthcare professionals and limited opportunities for carer involvement can hinder patients' ability to engage with and retain information about their medicines [44]. Patients may also feel reluctant to ‘trouble’ staff with questions, reinforcing passive involvement in decision‐making. Decision aids and written information have been shown to improve patient knowledge, involvement and satisfaction with care [35]. Future interventions should therefore incorporate accessible written resources and proactive clinician‐led questioning (e.g., ‘How do you feel about stopping this medicine?’) to better support patient and carer involvement. In addition, carers play a critical role in medication management, particularly for patients with cognitive impairment, yet are often underinvolved in hospital decision‐making [45].

It was feasible to collect clinical outcome data from patients; however, it is important to note that patients who lacked capacity to consent were not included, which represents a substantial proportion of the target population and may have implications for representativeness. Collecting outcomes from this group presents additional ethical and practical challenges. Clinical outcomes were exploratory. Functional measures, mood and quality of life outcomes did not show significant change, but changes in delirium scores and cognition at 3 months were observed. These findings should be interpreted cautiously, as they likely reflect the small sample size, heterogeneity in recovery following acute illness and the absence of a comparator group. Future trials should incorporate strategies to improve recruitment of patients with cognitive impairment, including the use of consultee or proxy consent procedures and greater involvement of carers in decision‐making, reflecting their important role in medication management. In addition, strategies to improve recruitment of patients with cognitive impairment, strengthen implementation fidelity and include longer follow‐up to better understand sustainability, safety and clinical impact, in line with recommendations for pilot and feasibility research [33].

Based on the findings of this feasibility study, future iterations of the intervention should: (1) integrate the digital tool within electronic health records; (2) implement a formal process for documenting medication reviews undertaken in response to digital prompts, including whether recommendations were reviewed and acted upon, to support assessment of intervention fidelity; (3) incorporate proxy/consultee consent procedures to support inclusion of patients lacking capacity; (4) increase involvement of carers in medication decision‐making; (5) provide accessible written information for patients and carers; and (6) improve communication between hospital and primary care to support sustained deprescribing after discharge.

### 4.1 Strengths and Limitations

This study has several notable strengths. The intervention was codesigned with geriatricians and pharmacists, ensuring clinical relevance, feasibility within real‐world workflows and strong stakeholder engagement from the outset. The mixed‐methods design enabled triangulation of quantitative feasibility and medication outcomes with qualitative insights into acceptability, providing a richer understanding of implementation processes and contextual factors influencing uptake. Outcome completion rates were excellent, demonstrating that collecting patient‐reported and clinical measures in this population is achievable, whereas follow‐up extending beyond hospital discharge allowed preliminary exploration of whether medication changes were sustained and provided insight into short‐term trajectories after acute illness.

However, several limitations should be considered when interpreting the findings. The small sample size and feasibility design limit statistical power and preclude firm conclusions about effectiveness or clinical outcomes, whereas the absence of a control group means observed changes cannot be attributed to the intervention alone and may reflect natural recovery following acute illness. A high proportion of ineligible patients, largely due to lack of capacity or rapid discharge raises concerns about representativeness and highlights challenges in recruiting a typical older inpatient population, potentially introducing selection bias towards fitter or less cognitively impaired individuals. Future research should consider strategies to include patients lacking capacity, as they represent a substantial proportion of the target population and may derive significant benefit from anticholinergic reduction. The study was also not powered to assess safety outcomes, rare adverse events or longer term outcomes, and follow‐up duration may have been insufficient. Although delivery of the prompt was recorded, no systematic data were collected on whether clinicians reviewed the prompt, undertook a medication review or acted on the recommendation, limiting assessment of intervention fidelity. Furthermore, reliance on manual prompts (stickers and clinician review) rather than fully integrated automated electronic alerts may have introduced variability in implementation fidelity and limits scalability without further digital integration. Finally, the single‐site design and predominantly homogeneous participant demographics may reduce generalisability to other healthcare systems or more diverse populations.

## 5 Conclusions

This feasibility study identified several important challenges associated with implementing a digitally supported deprescribing intervention targeting anticholinergic burden in hospitalised older adults. Recruitment proved particularly difficult, driven by the high prevalence of cognitive impairment among eligible patients and short hospital stays, which substantially limited the pool of participants able to consent. In addition, operational and workflow constraints affected consistent delivery of the intervention. Although those who were eligible engaged well and follow‐up was achievable, these findings highlight key feasibility limitations and the need for adapted recruitment approaches and improved integration into clinical workflows before progressing to a larger trial. The intervention was associated with modest reductions in anticholinergic burden and exploratory changes in cognition and delirium; however, these findings should be interpreted cautiously given the small sample size, lack of a control group and the potential influence of recovery following acute illness. As such, no conclusions can be drawn regarding effectiveness. Importantly, the study identified several implementation challenges, including technical limitations, workforce constraints and variability in intervention delivery, alongside broader system‐level issues such as fragmented communication and limited patient and carer involvement. These factors highlight the need for improved integration into clinical workflows, enhanced multidisciplinary collaboration and stronger continuity between hospital and primary care to support sustained deprescribing. Overall, this study provides valuable insights into the feasibility, acceptability and practical considerations of delivering a deprescribing intervention in acute care. Further research is warranted to optimise the intervention, improve inclusivity particularly for patients lacking capacity and evaluate clinical and cost‐effectiveness in a larger, controlled study.

## Author Contributions

E.R. and K.I. were responsible for undertaking the intervention development work and for conducting the feasibility study, in collaboration with all other authors. K.I. led the writing of the paper. All authors provided clinical and academic expertise and contributed to the development and writing of the paper. T.S. and S.M. led the health economic analysis. N.K. led the statistical analysis.

## Funding

This study is funded by the National Institute for Health and Care Research Applied Research Collaboration (ARC) Wessex. The views expressed in this publication are those of the authors and not necessarily those of the National Institute for Health and Care Research or the Department of Health and Social Care.

## Conflicts of Interest

The authors declare no conflicts of interest.

## Supporting information


**Appendix S1:** STROBE Statement—checklist of items that should be included in reports of observational studies.
**Appendix S2:** Health economic analysis.
**Table S1:** Unit costs.
**Table S2:** Mean (SD) resource use at baseline and 3‐month follow‐up (based on available data).
**Table S3:** Mean (SD) medication use at baseline, hospital discharge, one month, and three months (based on available cases).
**Table S4:** Mean (SD) costs at baseline and 3‐month follow‐up (based on available data).
**Table S5:** Mean (SD) medication costs at baseline, discharge, one month, and three months.
**Table S6:** Descriptive analysis of EQ‐5D‐5L responses at baseline and 3‐month follow‐up (available case data).
**Table S7:** Mean (SD) utility and QALYs at each time point (available case data).
**Appendix S3:** Qualitative interview topic guides.

## Data Availability

The data that supports the findings of this study are available in the supplementary material of this article
