# Supplementary material for: The Feasibility and Acceptability of a Collaborative Deprescribing Intervention to Reduce Anticholinergic Burden Among Hospitalised Older Patients
Source: Basic Clin Pharmacol Toxicol. 2026 Aug 23;139(3):e70277. doi: 10.1111/bcpt.70277 (PMC13501056; doi:10.1111/bcpt.70277)
Supplement: Supplementary file 1 [file BCPT-139-0-s001.docx]

Appendix S1: STROBE Statement—checklist of items that should be included in reports of observational studies

|  | Item No | | Recommendation | Page  No |
| --- | --- | --- | --- | --- |
| **Title and abstract** | 1 | | (*a*) Indicate the study’s design with a commonly used term in the title or the abstract | 1 |
|  |  |  | (*b*) Provide in the abstract an informative and balanced summary of what was done and what was found | 2 |
| Introduction | | | | |
| Background/rationale | 2 | | Explain the scientific background and rationale for the investigation being reported | 3 |
| Objectives | 3 | | State specific objectives, including any prespecified hypotheses | 3 |
| Methods | | | | |
| Study design | 4 | | Present key elements of study design early in the paper | 4 |
| Setting | 5 | | Describe the setting, locations, and relevant dates, including periods of recruitment, exposure, follow-up, and data collection | 4 |
| Participants | 6 | | (*a*) *Cohort study*—Give the eligibility criteria, and the sources and methods of selection of participants. Describe methods of follow-up  *Case-control study*—Give the eligibility criteria, and the sources and methods of case ascertainment and control selection. Give the rationale for the choice of cases and controls  *Cross-sectional study*—Give the eligibility criteria, and the sources and methods of selection of participants | 4 |
|  |  |  | (*b*) *Cohort study*—For matched studies, give matching criteria and number of exposed and unexposed  *Case-control study*—For matched studies, give matching criteria and the number of controls per case |  |
| Variables | | 7 | Clearly define all outcomes, exposures, predictors, potential confounders, and effect modifiers. Give diagnostic criteria, if applicable | 5 |
| Data sources/ measurement | | 8* | For each variable of interest, give sources of data and details of methods of assessment (measurement). Describe comparability of assessment methods if there is more than one group | *5* |
| Bias | | 9 | Describe any efforts to address potential sources of bias | 6 |
| Study size | | 10 | Explain how the study size was arrived at | 5 |
| Quantitative variables | | 11 | Explain how quantitative variables were handled in the analyses. If applicable, describe which groupings were chosen and why | 6 |
| Statistical methods | | 12 | (*a*) Describe all statistical methods, including those used to control for confounding | 6 |
|  |  |  | (*b*) Describe any methods used to examine subgroups and interactions | NA |
|  |  |  | (*c*) Explain how missing data were addressed | NA |
|  |  |  | (*d*) *Cohort study*—If applicable, explain how loss to follow-up was addressed  *Case-control study*—If applicable, explain how matching of cases and controls was addressed  *Cross-sectional study*—If applicable, describe analytical methods taking account of sampling strategy |  |
|  |  |  | (*e*) Describe any sensitivity analyses | NA |

Continued on next page

| Results | | | |
| --- | --- | --- | --- |
| Participants | 13* | (a) Report numbers of individuals at each stage of study—eg numbers potentially eligible, examined for eligibility, confirmed eligible, included in the study, completing follow-up, and analysed | 7 |
|  |  | (b) Give reasons for non-participation at each stage | 7 |
|  |  | (c) Consider use of a flow diagram | 7 |
| Descriptive data | 14* | (a) Give characteristics of study participants (eg demographic, clinical, social) and information on exposures and potential confounders | 8 |
|  |  | (b) Indicate number of participants with missing data for each variable of interest | 8 |
|  |  | (c) *Cohort study*—Summarise follow-up time (eg, average and total amount) | 8 |
| Outcome data | 15* | *Cohort study*—Report numbers of outcome events or summary measures over time | *8-9* |
|  |  | *Case-control study—*Report numbers in each exposure category, or summary measures of exposure |  |
|  |  | *Cross-sectional study—*Report numbers of outcome events or summary measures |  |
| Main results | 16 | (*a*) Give unadjusted estimates and, if applicable, confounder-adjusted estimates and their precision (eg, 95% confidence interval). Make clear which confounders were adjusted for and why they were included | 8-9 |
|  |  | (*b*) Report category boundaries when continuous variables were categorized | 8-9 |
|  |  | (*c*) If relevant, consider translating estimates of relative risk into absolute risk for a meaningful time period | NA |
| Other analyses | 17 | Report other analyses done—eg analyses of subgroups and interactions, and sensitivity analyses | 10-12 |
| Discussion | | | |
| Key results | 18 | Summarise key results with reference to study objectives | 12-13 |
| Limitations | 19 | Discuss limitations of the study, taking into account sources of potential bias or imprecision. Discuss both direction and magnitude of any potential bias | 14 |
| Interpretation | 20 | Give a cautious overall interpretation of results considering objectives, limitations, multiplicity of analyses, results from similar studies, and other relevant evidence | 15 |
| Generalisability | 21 | Discuss the generalisability (external validity) of the study results | 15 |
| Other information | | | |
| Funding | 22 | Give the source of funding and the role of the funders for the present study and, if applicable, for the original study on which the present article is based | 15 |

*Give information separately for cases and controls in case-control studies and, if applicable, for exposed and unexposed groups in cohort and cross-sectional studies.

**Note:** An Explanation and Elaboration article discusses each checklist item and gives methodological background and published examples of transparent reporting. The STROBE checklist is best used in conjunction with this article (freely available on the Web sites of PLoS Medicine at http://www.plosmedicine.org/, Annals of Internal Medicine at http://www.annals.org/, and Epidemiology at http://www.epidem.com/). Information on the STROBE Initiative is available at www.strobe-statement.org.

**Appendix S2: Health economic analysis**

**Table S1:** Unit costs

| **Resource Items** | **Unit Cost**  **(2023/24 £)** | **Source** |
| --- | --- | --- |
| **Intervention** | | |
| Screening patients using TRISCRIBE and placing a sticker on their notes, when completed by a registrar | £9.33 | PSSRU 23/24, assuming 10 minutes per patient as qualitatively estimated in the study |
| Screening patients using TRISCRIBE and placing a sticker on their notes, when completed by a hospital-based pharmacist | £9.17 | PSSRU 23/24, assuming 10 minutes per patient as qualitatively estimated in the study |
| **Primary and community care** | | |
| General Practitioner clinic visit | £37 | PSSRU 23/24 |
| General Practitioner virtual visit | £14.80 | PSSRU 23/24, assuming 5 minutes (based on length of time of GP-led telephone triage, PSSRU 23/24) |
| Practice pharmacist clinic visit | £9.50 | 23/24 PSSRU Band 6 1-hour unit cost, assuming 10 min visit |
| Practice pharmacist virtual visit | £9.50 | Assuming the same as a clinic visit as no separate cost data is available |
| Practice nurse clinic visit | £7.83 | PSSRU 23/24, assuming 10 min visit |
| Practice nurse virtual visit | £5.14 | Assuming 6.56 minutes (based on mean length of time of nurse-led telephone triage, PSSRU 23/24) |
| Other primary care clinic visit | £7.83 | Assuming an AoC band 5 professional who operates in a similar capacity to a nurse; PSSRU 23/24, assuming 10 min visit |
| Other primary care virtual visit | £5.14 | Assuming 6.56 minutes (based on mean length of time of nurse-led telephone triage, PSSRU 23/24) |
| NHS Walk in centre visit | £88 | NHS reference costs 23/24, weighted average of all walk in centre types |
| NHS out of hours visit | £273 | NHS reference costs 23/24. Weighted average of all emergency care |
| NHS 111 service (per call) | £13.80 | 2018/19 cost from linked paper adjusted for inflation to 2023/24 prices  [Cost–consequences of introducing NHS 111 Online - Impact of NHS 111 Online on the NHS 111 telephone service and urgent care system: a mixed-methods study - NCBI Bookshelf](https://www.ncbi.nlm.nih.gov/books/NBK575169/) |
| Health visitor in person or virtual visit | £64 | NHS reference costs 23/24, “Health visitor, other clinical intervention.” Virtual cost assumed to be the same because no virtual reference cost is given. |
| Physiotherapist in person or virtual visit | £41 | NHS reference costs 23/24. Virtual cost assumed to be the same because no virtual reference cost is given. |
| Occupational therapist in person or virtual visit | £68 | NHS reference costs 23/24. Virtual cost assumed to be the same because no virtual reference cost is given. |
| Dietician in person or virtual visit | £62 | NHS reference costs 23/24. Virtual cost assumed to be the same because no virtual reference cost is given. |
| Speech and language therapist in person or virtual visit | £100 | NHS reference costs 23/24, assuming one-to-one. Virtual cost assumed to be the same because no virtual reference cost is given. |
| Geriatric medical services in person or virtual visit | £161 | NHS reference costs 23/24. Integrated multidisciplinary team within Elderly Patients currency of Community Health Services. Virtual cost assumed to be the same because no virtual reference cost is given. |
| Frailty practitioner in person or virtual visit | £83 | NHS reference costs 23/24- long term conditions case management service within the elderly patients currency of community health services. Virtual cost assumed to be the same because no virtual reference cost is given. |
| Older persons specialist nurse in person visit | £116 | NHS reference costs 23/24- assume “Elderly patients” within district nursing service. Virtual cost assumed to be the same because no virtual reference cost is given. |
| Other community care visit in person visit | £86 | 23/24 NHS reference costs, weighted average of all community health services. Virtual cost assumed to be the same because no virtual reference cost is given. |
| **Secondary Care** | | |
| A&E visit | £143 | NHS reference costs- Weighted average of all “Consultant led mono specialty accident and emergency service” |
| Outpatient appointment in person visit | £186 | 23/24 NHS reference costs, weighted average of all F2F outpatient care (WF01A, WF01B, WF02A, WF02B) |
| Falls clinic in person visit | £352 | 23/24 NHS reference costs, consultant led Elderly Medicine Service F2F follow-up visit |
| Syncope clinic in person visit | £352 | 23/24 NHS reference costs, consultant led Elderly Medicine Service F2F follow-up visit |
| Other outpatient visit in person | £186 | 23/24 NHS reference costs, weighted average of all F2F outpatient care (WF01A, WF01B, WF02A, WF02B) |
| Virtual outpatient appointment | £145 | 23/24 NHS reference costs, weighted average of all non-F2F outpatient costs (WF01C, WF01D, WF02C, WF02D) |
| Falls clinic virtual appointment | £259 | 23/24 NHS reference costs, consultant led Elderly Medicine Service non-F2F follow-up visit |
| Syncope clinic virtual appointment | £259 | 23/24 NHS reference costs, consultant led Elderly Medicine Service non-F2F follow-up visit |
| Other virtual outpatient visit | £145 | 23/24 NHS reference costs, weighted average of all non-F2F outpatient costs (WF01C, WF01D, WF02C, WF02D) |
| Inpatient stay | £1,853 | NHS reference costs 23/24 weighted average of all admitted patient care |
| Medications | | |
| Prescribed medications (per medicine) | £9.01 | Average cost of everything on 23/24 PCA |

**Table S2: Mean (SD) resource use at baseline and 3-month follow-up (based on available data)**

| **Resource Items** | Baseline  Mean (SD) | N (%) | 3-month follow-up  Mean (SD) | N (%) | Mean (95% CI) difference |
| --- | --- | --- | --- | --- | --- |
| **Primary and community care (number of visits)** | | | | | |
| General Practitioner clinic visit | 0.80 (1.06) | 20 (100%) | 0.429 (0.514) | 14 (70%) | 0.071 (-0.350; 0.423) |
| General practitioner virtual visit | 0.10 (0.31) | 20 (100%) | 0.143 (0.363) | 14 (70%) | 0.071 (-0.203; 0.345) |
| Practice nurse clinic visit | 0.30 (0.73) | 20 (100%) | 0.50 (0.855) | 14 (70%) | 0.357 (-0.179; 0.893) |
| Practice nurse virtual visit | 0.05 (0.22) | 20 (100%) | 0 | 14 (70 %) | -0.071 (-0.226; 0.083) |
| Practice pharmacist clinic | 0.05 (0.22) | 20 (100%) | 0.071 (0.267) | 14 (70%) | 0 (0.392; -0.226) |
| Practice pharmacist virtual visit | 0 (0) | 20 (100%) | 0 (0) | 14 (70%) | 0 (0; 0) |
| Other primary care virtual visit | 0 (0) | 20 (100%) | 0 (0) | 14 (70%) | 0 (0; 0) |
| Other primary care in person visit | 0.30 (0.571) | 20 (100%) | 0.071 (0.267) | 14 (70%) | -0.143 (-0.451; 0.166) |
| NHS Walk in centre | 0.05 (0.224) | 20 (100%) | 0 (0) | 14 (70%) | -0.071 (-0.226; 0.083) |
| NHS out of hours visit | 0.05 (0.224) | 20 (100%) | 0 (0) | 14 (70%) | 0 (0, 0) |
| NHS 111 service ( call service) | 0.45 (0.945) | 20 (100%) | 0 (0) | 14 (70%) | -0.143 (-0.353; 0.067) |
| Health visitor (HV)- in person | 0 (0) | 20 (100%) | 0 (0) | 14 (70%) | 0 (0; 0) |
| Health visitor- virtual | 0 (0) | 20 (100%) | 0 (0) | 14 (70%) | 0 (0; 0) |
| Physiotherapist (PH)- in person | 0 (0) | 20 (100%) | 0.286 (0.825) | 14 (70%) | 0.286 (-0.191; 0.762) |
| Physiotherapist- virtual | 0 (0) | 20 (100%) | 0 (0) | 14 (70%) | 0 (0; 0) |
| Occupational therapist (OT )- in person | 0 (0) | 20 (100%) | 0.143 (0.535) | 14 (70%) | 0.143 (-0.166; 0.451) |
| Occupational therapist- virtual | 0 (0) | 20 (100%) | 0 (0) | 14 (70%) | 0 (0; 0) |
| Dietician (D)- in person | 0 (0) | 20 (100%) | 0 (0) | 14 (70%) | 0 (0; 0) |
| Dietician virtual | 0.05 (0.224) | 20 (100%) | 0 (0) | 14 (70%) | 0 (0; 0) |
| Speech and language (SLT)therapist- in person | 0.10 (0.447) | 20 (100%) | 0 (0) | 14 (70%) | 0 (0; 0) |
| Speech and language therapist virtual | 0 (0) | 20 (100%) | 0 (0) | 14 (70%) | 0 (0; 0) |
| Geriatric medical services (GMS)- in person | 0 (0) | 20 (100%) | 0 (0) | 14 (70%) | 0 (0; 0) |
| Geriatric medical services- virtual | 0 (0) | 20 (100%) | 0 (0) | 14 (70%) | 0 (0; 0) |
| Frailty practitioner (FP)- in person | 0.40 (1.789) | 20 (100%) | 0 (0) | 14 (70%) | 0 (0; 0) |
| Frailty practitioner- virtual | 0 (0) | 20 (100%) | 0 (0) | 14 (70%) | 0 (0; 0) |
| Older persons specialist (OPS)nurse- in person | 0 (0) | 20 (100%) | 0 (0) | 14 (70%) | 0 (0;0 ) |
| Older persons specialist nurse- virtual | 0 (0) | 20 (100%) | 0 (0) | 14 (70%) | 0 (0; 0) |
| Other community care- in person | 0.25 (0.550) | 20 (100%) | 0.357 (1.336) | 14 (70%) | 0 (-0.906; 0.906) |
| Other community care- virtual | 0.15 (0.366) | 20 (100%) | 0 (0) | 14 (70%) | -0.214 (-0.032; 0.460) |
| **Secondary Care (number of visits)** | | | | | |
| A&E | 0.80 (0.834) | 20 (100%) | 0 (0) | 14 (70%) | -0.786 (-1.30; -0.270) |
| Outpatient appointments- in person | 0.15 (0.366) | 20 (100%) | 0.071 (0.267) | 14 (70%) | -0.071 (-0.345; 0.203) |
| Outpatient appointments-virtual | 0 (0) | 20 (100%) | 0 (0) | 14 (70%) | 0 (0; 0) |
| Falls clinic- in person | 0 (0) | 20 (100%) | 0 (0) | 14 (70%) | 0 (0; 0) |
| Falls clinic- virtual | 0 (0) | 20 (100%) | 0 (0) | 14 (70%) | 0 (0; 0) |
| Syncope clinic- in person | 0 (0) | 20 (100%) | 0 (0) | 14 (70%) | 0 (0; 0) |
| Syncope clinic- virtual | 0 (0) | 20 (100%) | 0 (0) | 14 (70%) | 0 (0; 0) |
| Other secondary care- in person | 0.35 (0.745) | 20 (100%) | 0.357 (0.745) | 14 (70%) | 0 (-0.641; 0.641) |
| Other secondary care- virtual | 0.10 (0.308) | 20 (100%) | 0.071 (0.267) | 14 (70%) | -0.071 (-0.226; 0.083) |
| Inpatient visits | 1.10 (0.718) | 20 (100%) | 0.643 (0.842) | 14 (70%) | -0.571 (-1.240; 0.097) |
| **Total number of visits per patient** | 5.60 (4.08) | 20 (100%) | 3.14 (3.55) | 14 (70%) | -1.21 (-3.47; 1.04) |

**Table S3:** **Mean (SD) medication use at baseline, hospital discharge, one month, and three months (based on available cases)**

|  | Mean (SD) | N (%) | Mean (95% CI) difference compared to baseline |
| --- | --- | --- | --- |
| Medication count- Baseline | 14.35 (4.133) | 20 (100%) | N/A |
| Medication count- Discharge | 13.30 (3.785) | 20 (100%) | -1.05 (-2.454; 0.354) |
| Medication count- 1 month | 12.15 (4.694) | 20 (100%) | -2.2 (-3.854; -0.546) |
| Medication count- 3 months | 12.526 (4.526) | 19 (95%) | -1.789 (-3.432; -0.146) |

**Table S4: Mean (SD) costs at baseline and 3-month follow-up (based on available data)**

| **Resource Items** | Baseline  Mean(SD) | N (%) | 3-month follow-up  Mean (SD) | N (%) | Mean (95% CI) difference |
| --- | --- | --- | --- | --- | --- |
| Intervention costs | £10.00 (0) | 20 (100%) | £0 (0) | 20 (100%) | -£10.00 (-10; -10) |
| **Primary and community care (mean cost per patient)** | | | | | |
| General Practitioner clinic visit | £29.60 (139.08) | 20 (100%) | £15.86 (19.00) | 14 (70%) | £2.64 (-12.95; 18.24) |
| General practitioner virtual visit | £1.48 (4.56) | 20 (100%) | £2.11 (5.37) | 14 (70%) | £1.06 (-3.00; 5.11) |
| Practice nurse clinic visit | £2.35 (7.74) | 20 (100%) | £3.92 (6.69) | 14 (70%) | £2.80 ( -1.40; 7.00) |
| Practice nurse virtual visit | £0.26 (1.15) | 20 (100%) | £0 (0) | 14 (70%) | -£0.37 (-1.16; 0.43) |
| Practice pharmacist clinic | £0.48 (2.12) | 20 (100%) | £0.68 (2.54) | 14 (70%) | £0 (3.73; -2.15) |
| Practice pharmacist virtual visit | £0 (0) | 20 (100%) | £0 (0) | 14 (70%) | £0 (0; 0) |
| Other primary care virtual visit | £0 (0) | 20 (100%) | £0 (0) | 14 (70%) | £0 (0; 0) |
| Other primary care in person visit | £2.35 (4.47) | 20 (100%) | £0.56 (2.09) | 14 (70%) | -£1.12 (-3.54; 1.30) |
| NHS Walk in centre | £4.40 (19.68) | 20 (100%) | £0 (0) | 14 (70%) | -£6.29 (-19.87; 7.29) |
| NHS out of hours visit | £13.65 (61.04) | 20 (100%) | £0 (0) | 14 (70%) | £0 (0; 0) |
| NHS 111 service (call service) | £6.21 (13.03) | 20 (100%) | £0 (0) | 14 (70%) | -£1.97 (-4.86; 0.922) |
| Health visitor(HV)- in person | £0 (0) | 20 (100%) | £0 (0) | 14 (70%) | £0 (0; 0) |
| Health visitor- virtual | £0 (0) | 20 (100%) | £0 (0) | 14 (70%) | £0 (0; 0) |
| Physiotherapist (PH) in person | £0 (0) | 20 (100%) | £11.71 (33.84) | 14 (70%) | £11.71 (-7.83; 31.25) |
| Physiotherapist- virtual | £0 (0) | 20 (100%) | £0 (0) | 14 (70%) | £0 (0; 0) |
| Occupational therapist (OT)- in person | £0 (0) | 20 (100%) | £9.71 (36.35) | 14 (70%) | £9.71 (-11.27; 30.70) |
| Occupational therapist- virtual | £0 (0) | 20 (100%) | £0 (0) | 14 (70%) | £0 (0; 0) |
| Dietician (D)- in person | £0 (0) | 20 (100%) | £0 (0) | 14 (70%) | £0 (0; 0) |
| Dietician virtual | £3.10 (13.86) | 20 (100%) | £0 (0) | 14 (70%) | £0 (0; 0) |
| Speech and language (SLT)therapist- in person | £10.00 (44.72) | 20 (100%) | £0 (0) | 14 (70%) | £0 (0; 0) |
| Speech and language therapist virtual | £0 (0) | 20 (100%) | £0 (0) | 14 (70%) | £0 (0; 0) |
| Geriatric medical services (GMS)- in person | £0 (0) | 20 (100%) | £0 (0) | 14 (70%) | £0 (0; 0) |
| Geriatric medical services- virtual | £0 (0) | 20 (100%) | £0 (0) | 14 (70%) | £0 (0; 0) |
| Frailty practitioner (FP)- in person | £33.20 (148.47) | 20 (100%) | £0 (0) | 14 (70%) | £0 (0; 0) |
| Frailty practitioner- virtual | £0 (0) | 20 (100%) | £0 (0) | 14 (70%) | £0 (0; 0) |
| Older persons specialist (OPS)nurse- in person | £0 (0) | 20 (100%) | £0 (0) | 14 (70%) | £0 (0; 0) |
| Older persons specialist nurse- virtual | £0 (0) | 20 (100%) | £0 (0) | 14 (70%) | £0 (0; 0) |
| Other community care- in person | £21.50 (47.31) | 20 (100%) | £30.71 (114.92) | 14 (70%) | £0 (-77.91: 77.91) |
| Other community care- virtual | £12.90 (31.51) | 20 (100%) | £0 (0) | 14 (70%) | -£18.43 (-39.57; 2.72) |
| **Secondary Care (mean cost per patient)** | | | | | |
| A&E | £114.4 (119.19) | 20 (100%) | £0 (0) | 14 (70%) | -£112.36 (-£186.05; -38.66) |
| Outpatient appointments- in person | £27.90 (68.14) | 20 (100%) | £13.29 (49.71) | 14 (70%) | -£13.29 (-64.26; 37.69) |
| Outpatient appointments-virtual | £0 (0) | 20 (100%) | £0 (0) | 14 (70%) | £0 (0;0) |
| Falls clinic- in person | £0 (0) | 20 (100%) | £0 (0) | 14 (70%) | £0 (0;0) |
| Falls clinic- virtual | £0 (0) | 20 (100%) | £0 (0) | 14 (70%) | £0 (0;0) |
| Syncope clinic- in person | £0 (0) | 20 (100%) | £0 (0) | 14 (70%) | £0 (0;0) |
| Syncope clinic- virtual | £0 (0) | 20 (100%) | £0 (0) | 14 (70%) | £0 (0;0) |
| Other secondary care- in person | £65.1 (138.60) | 20 (100%) | £66.43 (138.56) | 14 (70%) | £0 (-119.14; 119.14) |
| Other secondary care- virtual | £14.50 (44.63) | 20 (100%) | £10.36 (38.75) | 14 (70%) | -£10.36 (-32.73; 12.02) |
| Inpatient visits | £2038.30 (1330.80) | 20 (100%) | £1191.21 (1560.04) | 14 (70%) | -£1058.86 (-2297.65; 179.94) |
| **Mean total visit and intervention cost per patient** | £2411.67 (1329.92) | 20 (100%) | £1356.55 (1621.77) | 14 (70%) | -£1205.10 (-2508.14; 97.93) |

**Table S5:** **Mean (SD) medication costs at baseline, discharge, one month, and three months**

|  | Mean (SD) | N (%) | Mean (95% CI) difference compared to baseline |
| --- | --- | --- | --- |
| Medication cost- Baseline | £129.29 (37.24) | 20 (100%) | N/A |
| Medication cost- Discharge | £119.83 (34.10) | 20 (100%) | -£9.46 (-22.11; 3.19) |
| Medication cost- 1 month | £109.47 (42.29) | 20 (100%) | -£19.82 (-34.72; -4.92) |
| Medication cost- 3 months | £112.86 (40.78) | 19 (95%) | -£16.12 (-30.93; -1.32) |

**Table S6: Descriptive analysis of EQ-5D-5L responses at baseline and 3-month follow-up (available case data)**

|  | **Baseline**  **(n =20)** | **3- month follow-up**  **(n=15)** |
| --- | --- | --- |
| **Baseline** | **Number (%)** | **Number (%)** |
| Mobility  1  2  3  4  5 | n=20  2 (10)  4 (20)  11 (55)  3 (15)  0 (0) | n=15  2 (13.33)  2 (13.33)  5 (33.33)  6 (40)  0 (0) |
| Self-care  1  2  3  4  5 | n=20  12 (60)  4 (20)  4 (20)  0 (0)  0 (0) | n=15  7 (46.67)  0 (0)  7 (46.67)  1 (6.67)  0 (0) |
| Usual activities  1  2  3  4  5 | n=20  7 (35)  8 (40)  3 (15)  1 (5)  1 (5) | n=15  5 (33.33)  4 (26.67)  4 (26.67)  1 (6.67)  1 (6.67) |
| Pain and discomfort  1  2  3  4  5 | n=20  6 (30)  6 (30)  5 (25)  2 (10)  1 (5) | n=15  2 (13.33)  3 (20)  4 (26.67)  6 (40)  0 (0) |
| Anxiety / Depression  1  2  3  4  5 | n=20  6 (30)  3 (15)  8 (40)  3 (15)  0 (0) | n=15  6 (40)  7 (46.67)  1 (6.67)  1 (6.67)  0 (0) |
| Number in perfect health (11111) | 0 (0%) | 0 (0%) |
| Number in worst health (55555) | 0 (0%) | 0 (0%) |
| Number of unique health states | 20 | 15 |

**Table S7: Mean (SD) utility and QALYs at each time point (available case data)**

|  | **Mean (sd)(Min, Max)** | **N (% of original sample)** |
| --- | --- | --- |
| **EQ-5D-5L Index** |  |  |
| **Baseline** | 0.587 (0.217)  (0.040, 0.868) | 20 (100%) |
| **3 Months** | 0.489 (0.272)  (0.031, 0.989) | 15 (75%) |
| **Unadjusted QALYs** | 0.128 (0.054)  (0.009, 0.228)* | 15 (75%) |
| **EQ-5D-5L VAS** |  |  |
| **Baseline** | 62.75 (25.3) (0, 95) | 20 (100%) |
| **3 Months** | 66.2 (24.04) (25, 98) | 15 (75%) |
| **Correlation between index and VAS** |  |  |
| **Baseline** | 0.447 (p=0.048) | 20 (100%) |
| **3 Months** | 0.427 (p=0.113) | 15 (75%) |

*Minimum value represents 3.6% of perfect health and maximum value represents 91.2% of perfect health over 3 months

Appendix S3. Qualitative interview topic guides

**A. Patient evaluation interview topic guide**

*You recently took part in a research study of an intervention to reduce potentially harmful medications during hospital admission. I would like to ask you a few questions about this and how you found it so we can understand what worked or did not work well and make any necessary improvements.*

**Opening question, attitudes and knowledge of medications**

First, I would like to understand your experience of daily management of your medicines.

How well do you feel you understand the reasons for taking each of your medicines?

What are the challenges you experience with taking your medication?

What strategies do you use to ensure you take your medicines correctly?

How well do you feel supported and listened to by your healthcare professional(s) when you have questions or concerns about your medicines? [If not] Why not? Can you give me a recent example that made you feel this way?

**The process of making changes to medicines**

Thinking back to when you were in hospital, you may have had changes made (eg reduction/ stopped) to one or more medications by your doctor or pharmacist. Can you please tell me whether any changes were made to your medicines?

What were these changes? Which medicines were changed? And what were the reasons for the changes?

How did the doctor or hospital pharmacist talk to you about the need for these changes and the reasons for them?

How did you feel the communication was between you and the Doctor/ pharmacist?

Did you feel you received enough information and support from your healthcare professional to make you feel comfortable about changing your medicines?

What went well and what could have gone better (during consultation)?

(If no changes were made to medication) please tell me in general about how you feel about taking your medications? What is your understanding of the reasons for taking each medication? How would you feel if any changes to your medication were suggested by a healthcare professional?

**Attitudes about medication changes**

Can you tell me how you felt about the decision to change some of your medicines? (Eg. relieved, anxious, reluctant, apprehensive?)

Did you have any concerns? What were they? Did you discuss these with the Doctor/ pharmacist?

How involved did you feel in the decision about your medicines? Were you given the opportunity to fully discuss your thoughts and explore your options? Did you feel able/ comfortable asking questions? Were any of your family members/ carers also involved in the decision?

How much did you feel your views and preferences were considered in decisions about your medicines?

**After hospital discharge**

Can you tell me about your experiences with medication management after being discharged from hospital?

Have you experienced any problems with medications (eg. any side effects, symptoms etc?)

Have the changes made during hospital admission been maintained after you came back home? If no, can you explain what happened?

Have any further changes been made to your medicines since you came out of hospital? What kind of changes have been made (eg. any medications stopped/started)? By whom and why?

Did you feel you needed support from a healthcare professional after your medicines were changed? And if so, did you feel you received it?

If (or when) you needed to contact a healthcare professional to discuss anything related to your medicines, do you know who to contact? Who would you contact in the first instance if you needed to ask questions or raise concerns? Why?

Could you tell me whether you have had any contact with your GP/ practice nurse/ practice pharmacist about your medicines since you came out of hospital? If so, please tell me about this. Were you contacted by anyone from your GP surgery? Why? How many times? Or did you contact the surgery yourself? And why?

If not, do you feel it is important for your healthcare professional to contact you about your medications after you came out of hospital?

**General questions**

Are there any improvements that could have been made to this process of making changes to your medicines whilst you were in hospital?

Is there anything else that is relevant to taking part in this research, that we haven’t discussed? If so, tell me about this.

B. **Carer evaluation interview topic guide**

*Your family member/ person you care for recently took part in a research study of an intervention to reduce potentially harmful medications during hospital admission. I would like to ask you a few questions about this and how you found it so we can understand what worked or did not work well and make any necessary improvements.*

**Opening question, attitudes and knowledge of medications**

First, I would like to understand your experience of management of the medicines of your family members/ person you care for.

Can you tell me about your role in managing the medicines of your family member/ person you care for?

How well do you feel you understand the reasons for them taking each of your medicines?

What are the challenges you experience with managing their medication?

What strategies do you use to ensure they take your medicines correctly?

Are you involved in the medical appointments about their medicines?

If so, how well do you feel supported and listened to by the healthcare professional(s) when you have questions or concerns about your medicines? [If not] Why not? Can you give me a recent example that made you feel this way?

**The process of making changes to medicines**

Thinking back to when your family member/ person you care for was in hospital, they may have had changes made (eg reduction/ stopped) to one or more medications by their doctor or pharmacist. Can you please tell me whether any changes were made to their medicines?

What were these changes? Which medicines were changed? And what were the reasons for the changes?

How did the doctor or hospital pharmacist talk to you and/ or your family member/person you care for about the need for these changes and the reasons for them?

How did you feel the communication was between you/ your family member/ the person you care for and the Doctor/ pharmacist?

Did you feel you/ they received enough information and support from your healthcare professional to make you/ them feel comfortable about changing your medicines?

What went well and what could have gone better (during consultation)?

(If no changes were made to medication) please tell me in general about how you feel about them taking medications? What is your understanding of the reasons for taking each medication? How would you feel if any changes to the medication were suggested by a healthcare professional?

**Attitudes about medication changes**

Can you tell me how you felt about the decision to change some of their medicines? (Eg. relieved, anxious, reluctant, apprehensive?)

Did you have any concerns? What were they? Did you discuss these with the Doctor/ pharmacist?

How involved did you feel, as a family member/carer in the decision about your medicines? Were you given the opportunity to fully discuss your thoughts and explore your options? Did you feel able/ comfortable asking questions? Were any other family members/ carers also involved in the decision?

How much did you feel your/ their views and preferences were considered in decisions about your medicines?

**After hospital discharge**

Can you tell me about your experiences with medication management after your family member/ person you care for was discharged from hospital?

Have they experienced any problems with the medications (eg. any side effects, symptoms etc?)

Have the changes made during hospital admission been maintained after they came back home? If no, can you explain what happened?

Have any further changes been made to their medicines since you came out of hospital? What kind of changes have been made (eg. any medications stopped/started)? By whom and why?

Did you feel you needed support from a healthcare professional after their medicines were changed? And if so, did you feel you received it?

If (or when) you needed to contact a healthcare professional to discuss anything related to their medicines, do you know who to contact? Who would you contact in the first instance if you needed to ask questions or raise concerns? Why?

Could you tell me whether you have had any contact with your GP/ practice nurse/ practice pharmacist about their medicines since you came out of hospital? If so, please tell me about this. Were you contacted by anyone from your GP surgery? Why? How many times? Or did you contact the surgery yourself? And why?

If not, do you feel it is important for their healthcare professional to contact you/ them about your medications after you came out of hospital?

**General questions**

Are there any improvements that could have been made to this process of making changes to their medicines whilst you were in hospital?

Is there anything else that is relevant to taking part in this research, that we haven’t discussed? If so, tell me about this.

C. **Hospital staff evaluation interview topic guide:**

*Thank you for taking part in delivering an intervention, aiming to reduce potentially harmful medications during hospital admission through using a digital tool to identify patients with a high score of anticholinergic burden. We would like to ask you some questions on your experience of this and any suggestions you may have for improvement.*

**Opening questions, views about anticholinergic medications**

First I would like to understand how important you feel it is to identify patients taking multiple medications with high anticholinergic side effects?

In your experience, how easy or difficult is it to reduce anticholinergic burden in older people with multimorbidity who are taking regular multiple medications?

**Intervention delivery**

*I would now like to move on to ask you questions about the process of using TRISCRIBE ACB dashboard to identify patients with high ACB scores and the outcomes of that on patients care.*

Please tell me about your role in the delivery of the DART intervention?

Did you use the TRISCRIBE tool to identify patients with a high ACB burden? If yes,

- Which staff used the TRSICRIBE tool? (E.g. pharmacists, pharmacy technician, junior doctors, consultants?
- How did the TRISCRIBE tool fit in the daily routine of staff?
- What are your views and experiences of using the TRISCRIBE tool?
- What do you think is the value and benefits of integrating this tool in routine practice?
- Were there any challenges you faced in using the tool?

If not, are you aware of the TRISCRIBE ACB tool? What do you think of it? In your opinion, who should be using the tool?

Please tell me about how this process may differ from usual care for older patients admitted to MOP in relation to reviewing and making any necessary changes to their medicines?

Now can talk me through what happened after patients were identified to have high risk of anticholinergic burden?

How was this information communicated with the clinical team on the wards? What strategies were used (note sticker, verbally during the ward rounds, other ways?)

What information was communicated to the clinical team on the wards? (e.g., ACB total score, types of ACB medications, recommendations for stopping or switching to safer alternatives?

Who was this information targeted to? (e.g. Consultants or junior doctors)? And how did they used the information to make a decision about whether or not to change medications prescribed?

What additional (if any) resources were used to support decision making? (training, teaching, other materials)?

**Information provision and communication with patients and General Practices**

Please tell me about how any information about ACB score and the need for potential changes to medicines, and the reasons for these, were communicated to patients? Were there any challenges with this?

What support did you provide the patient to make him/her feel comfortable about changing their medicines? What went well and what could have gone better?

Were patients and/ or family members/ carers involved in the decision process? How were they involved? How did you ensure that patients' priorities were taken into consideration? Can you give me an example of when this happened?

Did the patient have any concerns during the discussion about the suggested medication changes? What were these concerns? And how was the decision made?

How often do patients agree or disagree with a decision you make about their medicines? [If so] Can you talk about an example of this and what the outcome was?

How were changes to medicines communicated to the patient’s General Practice?

What specific information was communicated to the patient’s General Practice?

How does this differ to the usual process for communicating with General Practices?

**The impact of medication changes**

How easy or difficult was it to make decisions to reduce or stop anticholinergic medications? What do you think makes it difficult for you? Are there any aspects which may facilitate this process?

Please tell me how you monitor patients who have changes made to their medications during hospital admission and after discharge?

**General questions**

How would you describe your overall views and experience of the intervention? Any challenges or positive outputs you could think of?

In your experience, how practical was this intervention to carry out?

In general, tell me about what worked well with the intervention?

In general, tell me about what did not work so well with the intervention?

Were there any challenges you faced in delivering the intervention?

Are there any improvements at all that could be made to any stages of the DART intervention?
